# MultiMap: A Tool to Automatically Extract and Analyse Spatial Microscopic Data From Large Stacks of Confocal Microscopy Images

**DOI:** 10.3389/fnana.2018.00037

**Published:** 2018-05-23

**Authors:** Gherardo Varando, Ruth Benavides-Piccione, Alberto Muñoz, Asta Kastanauskaite, Concha Bielza, Pedro Larrañaga, Javier DeFelipe

**Affiliations:** ^1^Computational Intelligence Group, Department of Artificial Intelligence, Universidad Politécnica de Madrid, Madrid, Spain; ^2^Departamento de Neurobiología Funcional y de Sistemas, Instituto Cajal (CSIC), Madrid, Spain; ^3^Laboratorio Cajal de Circuitos Corticales, Centro de Tecnología Biomédica, Universidad Politécnica de Madrid, Madrid, Spain; ^4^Departamento de Biología Celular, Universidad Complutense, Madrid, Spain

**Keywords:** segmentation, object detection, fluorescent image, puncta segmentation, vglut1, vgat, brain atlas, ImageJ

## Abstract

The development of 3D visualization and reconstruction methods to analyse microscopic structures at different levels of resolutions is of great importance to define brain microorganization and connectivity. MultiMap is a new tool that allows the visualization, 3D segmentation and quantification of fluorescent structures selectively in the neuropil from large stacks of confocal microscopy images. The major contribution of this tool is the posibility to easily navigate and create regions of interest of any shape and size within a large brain area that will be automatically 3D segmented and quantified to determine the density of puncta in the neuropil. As a proof of concept, we focused on the analysis of glutamatergic and GABAergic presynaptic axon terminals in the mouse hippocampal region to demonstrate its use as a tool to provide putative excitatory and inhibitory synaptic maps. The segmentation and quantification method has been validated over expert labeled images of the mouse hippocampus and over two benchmark datasets, obtaining comparable results to the expert detections.

## 1. Introduction

The brain works as a whole and it is well established that the principles of structural design (spatial distribution, number and types of neurons, and synapses per volume, etc.) and functional organization differ considerably in the different parts of the nervous system (DeFelipe, 2015). Therefore, one of the first steps toward understanding how brain circuits contribute to the functional organization of the brain is to integrate neuroanatomical information with genetic, molecular and physiological data in brain atlases. This integration would allow the generation of models that present the data in a form that can be used to reason, make predictions and suggest new hypotheses to discover new aspects of the structural and functional organization of the brain (e.g., Kleinfeld et al., 2011; da Costa and Martin, 2013; Egger et al., 2014; Markram et al., 2015; for a review see DeFelipe, 2017).

In the field of neuroanatomy, the use of classical techniques and the introduction of new procedures and powerful tools to examine the organization of the nervous system (reviewed in Jones, 2007; Smith, 2007; DeFelipe, 2010, 2017; Kleinfeld et al., 2011; Osten and Margrie, 2013) have allowed a noticeable enlargement of the acquisition of data, generating a high volume of complex data to be examined and interpreted. Thus, the development of 3D visualization and reconstruction methods to analyse structures at different levels is of great importance as the large volume of data generated is critical to define brain connectivity and function.

In the present study we developed a tool, called MultiMap, that allows the visualization, easy navigation, creation of regions of interest of any shape and size within a large brain area, 3D segmentation and quantification of fluorescent structures from large stacks of confocal microscopy images. We implement in MultiMap [through ImageJ (Schneider et al., 2012; Schindelin et al., 2015)] a new approach to deal with fast detection and counting of fluorescent puncta over large 3D stacks of confocal images with a low signal-to-noise ratio, containing several thousands of objects. As a proof of concept, we focused on the analysis of glutamatergic and GABAergic synapses which represent the majority of synaptic types in the brain (Peters and Palay, 1996). Specifically, we used a genetically modified mouse that expresses the flourescent protein vGlut-1 to label glutamatergic terminals (Alonso-Nanclares et al., 2004; Herzog et al., 2011), whereas to identify the GABAergic synapses we used immunocytochemistry for the vesicular GABA transporter (vGAT) known to correspond to GABAergic inhibitory axon terminals (Chaudhry et al., 1998; Minelli et al., 2003). Since most synapses are found in the neuropil (the gray matter regions between neuronal and glial somata and blood vessels which is made up mainly of axonal, dendritic and glial processes) the vast majority of synaptic markers (puncta) are found in the neuropil. Importantly, the structures and amount of neuropil varies considerably, depending not only on the brain region and subregions (i.e., different subfields, layers, etc.) but also in any given region (for example, between the fundus of a sulcus and the crown of a gyrus). Even within the same subregions, the amount of neuropil varies between section to section. Thus, it is critical to determine the amount of neuropil in order to compare the density of puncta between different subjects or experimental conditions. In confocal images labeled for the above synaptic markers, the cell bodies, the blood vessels, the fiber tracts and the section artifacts (such as incomplete or fragmented histological sections) appear in the images as almost black structures that for simplicity we call “holes.” Therefore, a major aim of the present study was to detect these holes automatically to avoid their inclusion in the estimation of the density of puncta.

Regarding visualization, there are several software tools that allow visualizing microanatomical details from stacks of confocal microscopy images [i.e., Imaris software (Bitplane), Neurolucida (Microbrighfield), ImageJ (Schneider et al., 2012; Schindelin et al., 2015)]. However, they only include the possibility to create a right-angled region of interest or do not support large (>20 GB) tile scan stacks of images. MultiMap displays, as a main advantage, the possibility to easily navigate within a large brain area and create one or several regions of interest without any restrictions on their shapes. Additionally, it allows for the creation and visualization of several working layers storing different kind of information (e.g., visual, calibration, regions of interest, clouds of points, pixel-wise information and markers).

Regarding sementation and quantification of fluorescent structures, several methods in 2D and 3D exist. Even if the development of automatic methods for object detection has been revealed impelling in neuroscience, the current state of the art in 3D automatic object detection and counting does not present definite solutions. To the best of our knowledge the majority of 3D object detection and counting algorithms are designed for cells' counting (see Schmitz et al., 2014 for a review), which are relatively easier to quantify. However, regarding the estimation of fluorescent puncta, the available methods have some limitations. What follows is a brief description of the methods available in the literature.

Fish et al. (2008) use an iterative procedure using thresholding and morphological segmentation. The 3D nature of the problem does not allow the use of density estimation techniques such as the density estimation without detection implemented in Ilastik (Sommer et al., 2011) and described in Fiaschi et al. (2012); in our case it is important to actually detect the objects, since a single punctum can appear in multiples slices of the stack while the sizes, orientations and distribution of the objects are not known a priori. Others methods such as those presented in Sturt and Bamber (2012) and Dumitriu et al. (2012) are not fully automatic and require an expert (e.g., to choose an appropriate threshold of the image). Moreover, they rely on thresholding, a global procedure that cannot be applied uniformly over different stacks where objects could be imaged at different intensity. In Heck et al. (2014) an automated 3D detection method of synaptic contacts is described. The segmentation is obtained with a region growing algorithm, using local maxima as seeds. The method obtains very good results over images with high signal-to-noise ratio but it is not able to deal with low signal-to-noise ratio and not deconvolved images. Danielson and Lee (2014) developed SynPAnal, a software for semi-automatic quantification of density and intensity of puncta from flourescence microscopy images that operates only on 2D. 3D stacks are projected via a maximum operator along the *z*-axis thus losing the 3D information. Also, despite the fact that 3D light microscopic techniques are limited to a lower level of resolution, they remain the method of choice to obtain large-scale spatial information regarding the number and the size of presynaptic terminals in large brain regions, in order to know the number of chemically identified synapses and provide putative synaptic functional parameters. Having in mind that we will apply the method to sets of various stacks, a key issue is to develop a fast algorithm, with a simple parametrization and without the need of being trained over expert segmentations. Briefly, the present method includes a segmentation procedure using maximum of Laplacians of Gaussian filter. Thereafter, an automated object detection process is performed using a fast tag propagation algorithm.

Maps created with MultiMap can be easily shared and published as standalone visualizations on simple web pages, simplifying enormously collaborations and the communications of relevant findings.

The paper is organized as follows: section 2.1 describes the data acquisition procedure. In section 2.2 we present the MultiMap software. In section 2.3 we describe the novel algorithm to detect and count objects. In section 2.4 we describe how we detect the neuropil and in section 2.5 the estimation of the puncta volume number densities is described. In section 2.6 the validation of the object detection is described. We present our conclusion and some future directions in section 3.

## 2. Methods and results

### 2.1. Data acquisition and preparation

#### 2.1.1. Tissue preparation

**Figure 1:**
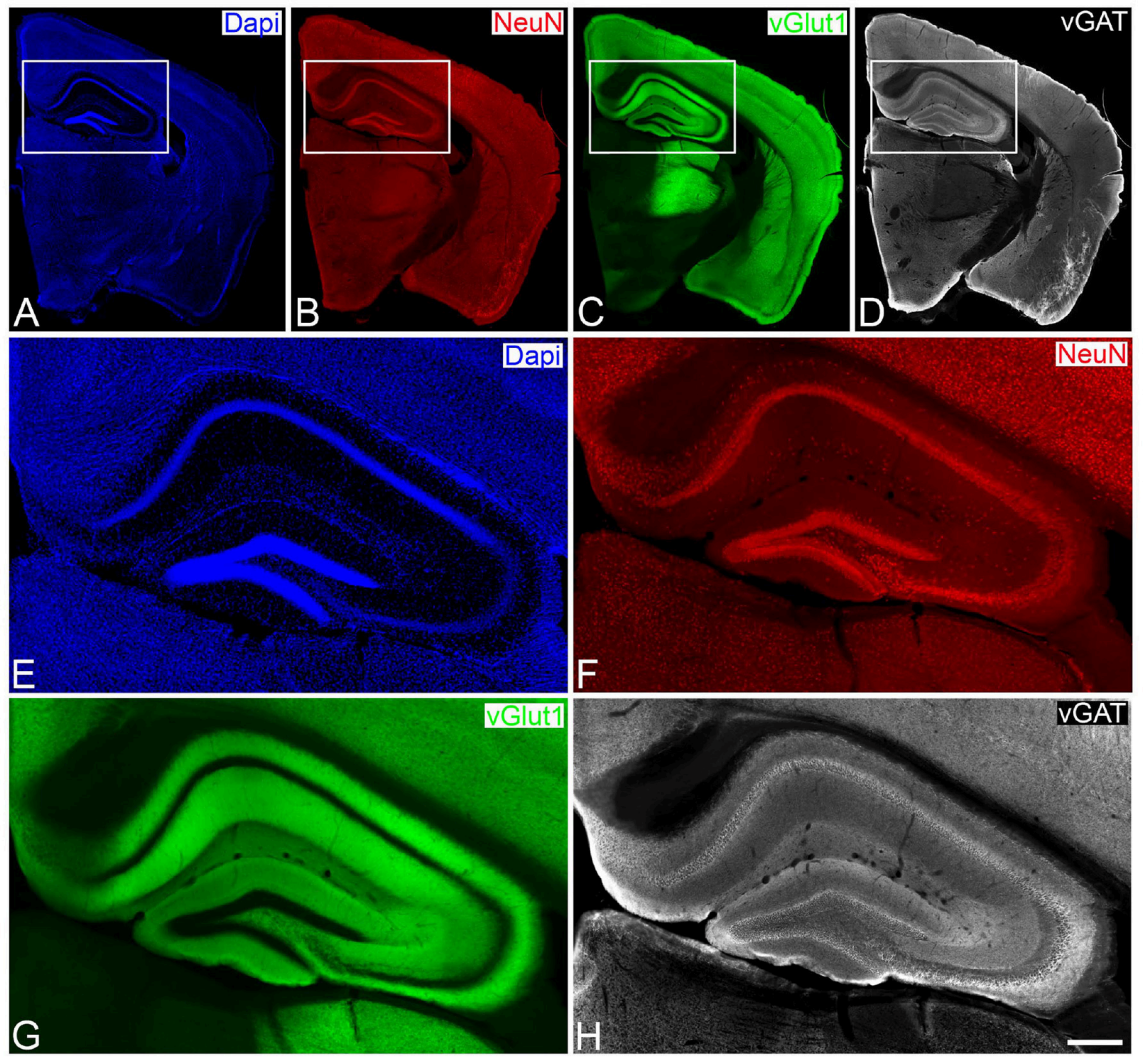
Low- **(A–D)** and higher- **(E–H)** power photomicrographs obtained with a conventional fluorescence microscope (tile scan; 10x objective), showing patterns of Dapi staining, and NeuN and vGat immunostaining of a brain section from a vGlut-1-transgenic mouse. The hippocampal region, squared zones in **A–D**, is shown at higher magnification in **E–H**, respectively. Scale bar (in **H**): 1,200μm in **A,B**; 275 μm in **E–H**.

Genetically modified VGLUT Venus Knock-in mice (vGlut-1) adult male mice (*n* = 6; 2 months old) were sacrificed by lethal intraperitoneal injection of sodium pentobarbital and then intracardially perfused with saline solution, followed by 4% paraformaldehyde in sodium phosphate buffer (PB: 0.1 M, pH 7.4) at room temperature. The brains were extracted from the skull and post-fixed in the same fixative overnight at 4 °C. After washing in PB, coronal vibratome sections of the brains (150 μm thick) were then cut with a Vibratome. For immunofluorescence, sections were preincubated for 1 h at room temperature in a stock solution containing 3% normal goat serum (Vector Laboratories, Burlingame, CA) in PB with Triton X-100 (0.25%). Then, sections were incubated in the same stock solution with rabbit anti-vGat (1:2,000, Synaptic Systems, Germany) and mouse-anti NeuN (1:2,000, Chemicon) antibodies that recognize vesicles in GABAergic axon terminals and neuronal cell bodies (to identify and classify laminar and cytoarchitectonic structures), respectively (Figure 1). After rinsing in PB, the sections were incubated for 2 h at room temperature in biotinylated goat anti-rabbit antibodies (1:200 Vector laboratories), rinsed in PB and then incubated with Alexa 594-coupled goat anti-mouse antibodies and Alexa 647-coupled streptavidin (1:2000 Molecular Probes). After rinsing sections were stained with the nuclear stain DAPI (4,6 diamidino-2-phenylindole; Sigma, St. Louis, MO, U.S.A.) that label cell nuclei (neurons, glia and perivascular cells) and then mounted in ProLong antifade mounting medium (Life Technologies).

#### 2.1.2. Confocal laser microscopy

Sections were then directly analyzed with the aid of a Zeiss LSM 710 Confocal microscope. Fluorescently labeled profiles were examined through separate channels, using excitation peaks of 401, 488, 594, and 634 nm to visualize DAPI, vGlut-1, NeuN, and vGat, respectively. Consecutive stacks of images, at high magnification (63×; oil immersion), using tile scan mode, were acquired in all layers of the hippocampal region, including the molecular layer, the granular cell layer and the polymorphic cell layer of the dentate gyrus, the stratum oriens, the pyramidal cell layer, the stratum radiatum and the stratum lacunosum-moleculare of the hippocampal subfields CA1, CA2, and CA3. Each stack was composed by images (0.14 μm z-step) of 1, 024 × 1, 024 pixels (8 bit). Confocal parameters were set so that the fluorescence signal was as bright as possible while ensuring that there were no saturated pixels. A total of 2,658 stacks of 15 images, 44 × 28 stacks for the vGlut-1 marker (Figure 1G) and 55 × 32 stacks for the vGat marker (Figure 1H), were acquired comprising approximately a surface of 4 mm^2 of tissue and over 47 GiB of data. The stack of images were processed with MultiMap and two interactive maps were created.

### 2.2. User interface

MultiMap is developed as a basic graphical user interface (GUI) and distributed as an open-source multi-platform desktop application. The main goal of MultiMap is to create and visualize *maps* anchoring spatial data (e.g., point clouds and densities), allowing the easy navigation and selection of regions of interest (ROI) within a large brain area from consecutive stacks of confocal microscopy images. We developed MultiMap as a modular application with the capabilities to use externals programs and libraries to perform specific tasks (e.g., ImageJ Schneider et al., 2012 for image analysis). In the following sections we explain the basic capabilities of MultiMap, see the online user guide for further explanations and tutorials^1^. An example map can be downloaded from the Cajal Blue Brain website^2^.

#### 2.2.1. Maps management

**Figure 2:**
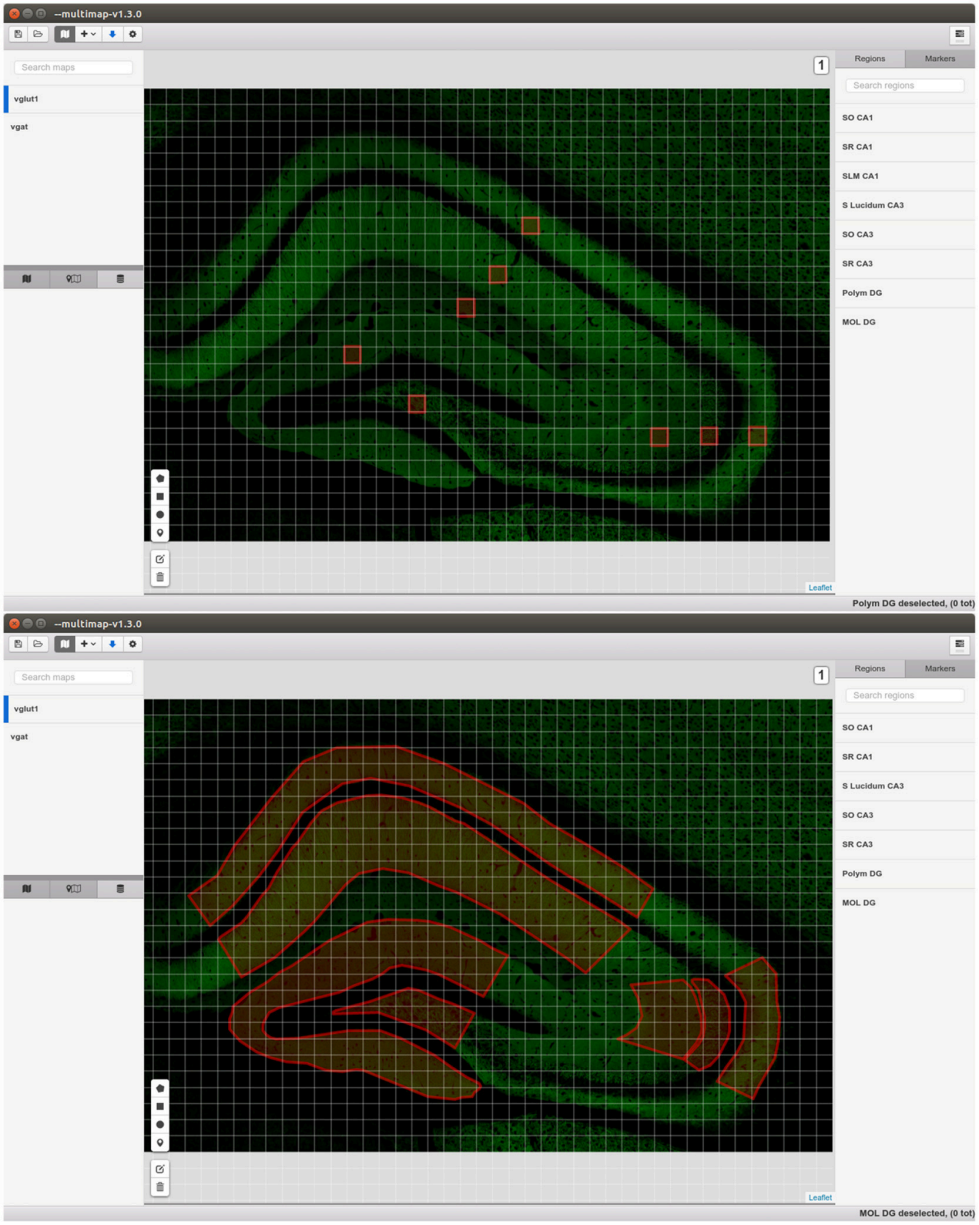
Graphical Interface of MultiMap.

The core of MultiMap can display, create, modify, load and export *maps* comprising several layers storing different kind of information: visual, calibration, regions of interest, clouds of points, pixel-wise information and markers. The maps display implementation (Figure 2) is based on *Leaflet*^3^, the leading open-source JavaScript library for interactive maps.

The information of every map (*map configuration*) is stored in a *object*, that is a collection of name/value pairs. We thus developed a leaflet plugin^4^ capable of creating all the layers from a *map configuration* object that we can store as a JSON file^5^. The map configuration object contains a *layers* field that stores the configurations of all the layers (*layer configuration* objects) in the map. While the map configuration object can be saved as a simple JSON file, each layer information (e.g., the actual images or the points coordinates) can be stored locally on the user machine or remotely, the layer configuration object has all the information needed to retrieve such information when needed (e.g., when displaying the images or when counting the points).

The user can easily create an empty map, add any type of layer and eventually export the configuration object as a JSON file directly from MultiMap.

#### 2.2.2. Layers type

We describe now the main types of layers^6^ that can be added to the map, thus the types of information that can be stored, visualized and analyzed.

- Graphical map tiles, that is “bitmap graphics displayed in a grid arrangement”^7^. The actual images can be stored both locally in the user machine, and remotely. They represent the visual information and allow the visualization of very large images since just few tiles are rendered. Since the output of confocal microscopy are stacks of images, MultiMap can visualize maps with different *levels* (that is *slices*), the user can easily switch between levels.
- ROIs and Markers can be drawn directly by the user in the application. They are saved and exported in the map configuration files allowing easily sharing of results and information.
- Layers of tiled points are used to visualize cloud of points stored in tiled csv files (locally or remotely). They permit to handle millions of points tiled across thousands of files.

#### 2.2.3. Density estimation and statistics

MultiMap can compute density estimations of points in ROIs and compute some simple statistics. For each selected region it is possible to compute the number of points included in the region, the density over the area or over the volume represented. The user can then export the results as a csv file, or display them directly in the application.

#### 2.2.4. Imagej integration

MultiMap is shipped with an extension that permits to use ImageJ (Schneider et al., 2012) for some image analysis and manipulation tasks. It is possible to perform the object detection algorithm (see section 2.3) and the holes detection workflow (see section 2.4) to automatically obtain the 3D segmentation and quantification of the elements present in the selected brain areas. Bio-Formats^8^ plugin can be used to convert images and it is possible to build graphical map tiles from any image^9^.

### 2.3. Object detection method

The object detection algorithm developed here receives as input a raw stack of images and produces as output a map of objects detected and a list of object centroids. The algorithm we designed is a combination of simple steps derived from classical image analysis algorithms combined with a novel one for objects tagging. The aim of the algorithm is to be fast, robust to noise and intensity changes, and able to handle big stacks of confocal images. The complete work flow is implemented as an independent ImageJ toolset^10^ and moreover is accessible from MultiMap via the ImageJ extension menu.

**Figure 3:**
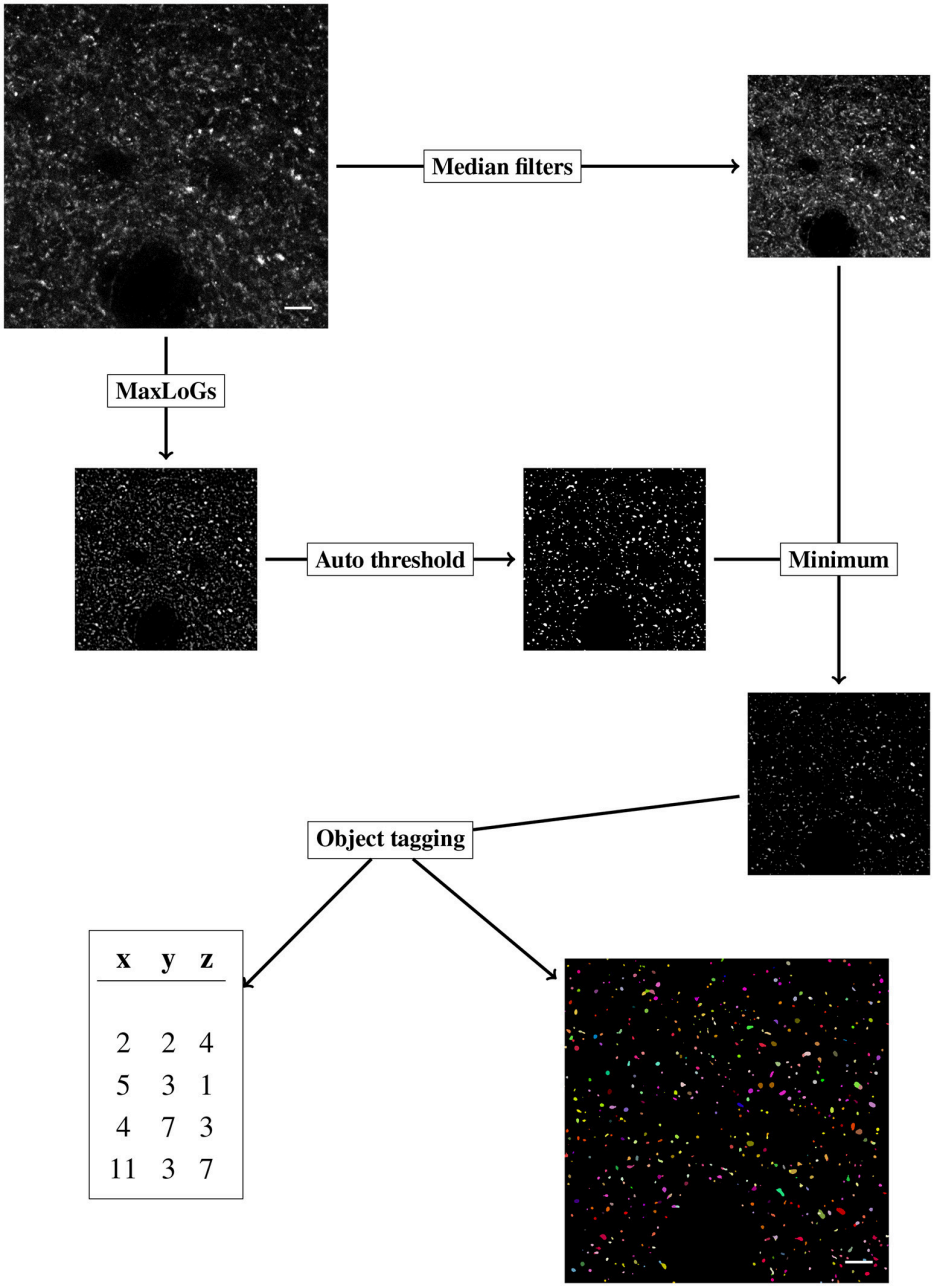
Diagram of the objects detection workflow, the steps of the algorithms are labeled in rectangular boxes. Scale bar is 5 μm and pixel intensity is enhanced for visualization.

We will denote by *I*(*x, y, z*) the intensity of the stack of images at pixel (*x, y, z*), where *x, y* represent the coordinates in the plane of every slice of the stack and *z* is the coordinate in the dimension of the stack depth. Next we list step by step the work-flow, a schematic representation is pictured in Figure 3.

The original stack of images is initially duplicated. The segmentation procedure (section-2.3.1) and the denoising phase (section-2.3.2) are performed independently on the copies of the original stack. The results of the segmentation (binary mask) and the denoised stack are then joined (pixel-wise minimum) and the result is used to perform the object tagging (section-2.3.3).

#### 2.3.1. Segmentation

For our purpose, segmentation is considered the task of producing a binary mask, differentiating background and foreground pixels. We employ a classical method of blob detection based on Laplacian-of-Gaussian filter (LoG) (Lindeberg, 1994). The method is robust to noise and fast to apply. LoG filter is obtained by convolving every image in the stack with a 2D Gaussian kernel with standard deviation σ, and successively applying the Laplacian operator. The LoG filter outputs a strong response for bright blobs of radius *r* approximately equal to $\sqrt{2}\sigma$. We thus indicate with LoG(*r*) the LoG filter with $\sigma =\frac{r}{\sqrt{2}}$. LoG(*r*) filter is sensible to the choice of the scale parameter *r*. To obtain a less scale-dependent filter we apply the LoG(*r*) with *r* varying in a given range of values (from *r*_*min*_ to *r*_*max*_ by steps of length δ) and for every pixel we select the maximum response among the computed ones. We indicate with MaxLoGs(*r*_*min*_, *r*_*max*_, δ) the result of the above procedure. Intuitively the MaxLoGs(*r*_*min*_, *r*_*max*_, δ) filter finds blobs of size varying approximately from *r*_*min*_ to *r*_*max*_.

The MaxLoGs filter is implemented in Java as an ImageJ plugin.^11^

Successively the output of the MaxLoGs(*r*_*min*_, *r*_*max*_, δ) filter is automatically converted into a binary mask using an automatic method (*thrMethod*). We found that the moments preserving algorithm (Glasbey, 1993) was the best suited to the fluorescent puncta detection.

#### 2.3.2. Denoising

We apply two-scale median filter (radius 1 and 2) to remove noise from the original stack while preserving edges (Arias-Castro and Donoho, 2009). Next we apply the mask obtained with the MaxLoGs filter retaining the foreground pixels and setting to zero the background ones. This step is achieved by computing the pixel-wise minimum between the binary mask and the denoised image.

#### 2.3.3. Object tagging

Once the foreground and background pixels have been detected, we proceed to tag the objects with an automating algorithm. We wish to associate every foreground pixel in the stack with a positive integer *i*, indicating that the given pixel belongs to the *i*-th object. The procedure is divided into three steps: slice tagging, slice connection and size checking.

- **Slice tagging:** Independently on every slice, we identify connected objects by nearest-neighbor propagation of object tags. Specifically, we start at a foreground pixel that has not been tagged, say (*x, y*). We tag the pixel with a new positive integer and we propagate the number to every (tagged or untagged) nearest pixels that have a positive intensity less or equal to the intensity of (*x, y*) plus a positive tolerance parameter (*toll*). From the new tagged pixels we keep propagating the number until no further propagation is possible. We move to a new, untagged pixel and we repeat. Since the propagation can overwrite the already tagged pixels we deduce that the results obtained are equivalent to propagate tags from the relative maxima of the intensity. We obtain in every slice a complete tagging of every foreground pixel in distinct objects.
- **Slice connection:** We connect the objects in different slices basing on the Bhattacharyya coefficient (Bhattacharyya, 1946). In particular, for every pair of objects *i* and *j* on adjacent slices we compute the Bhattacharyya coefficient between the distribution of the intensity of two objects, that is,
  $$BC\left(i,j\right)=\underset{\left(x,y\right)}{\sum }\sqrt{NI\left(i,x,y\right)NI\left(j,x,y\right)},$$
  where *NI*(*i, x, y*) is the normalized intensity of object *i* in the pixel (*x, y*),
  $$NI\left(i,x,y\right)=\frac{I\left(x,y,z\right)}{\sum_{\left(t,q,s\right)\in \text{object }i}I\left(t,q,s\right)}.$$
  If the Bhattacharyya coefficient between two objects surpasses a given parameter, ϕ, we join the two objects and tag them with a unique number accordingly.
- **Sizes checking:** In this last phase we check the size in pixels of the tagged objects. If the size of an object, in pixels, is less than a given parameter (*minSize*) we try to assign the object's pixels to adjacent objects. If no adjacent objects are found we assign the pixels to the background.

The final output of the algorithm is a stack of the same dimension as the original, where every pixel has the value 0 if it is a background pixel or it carries the positive integer tag representing the object that the pixel belongs to. Moreover, the algorithm computes, for every object detected, the centroid, that is, the average of the object's pixels coordinates, the volume and the surface area.

For further stereology-based analysis and estimations (see section 2.5), centroids of objects touching the borders of the stack are properly identified. In particular in the centroid list we specify, for every centroid, if its corresponding object is touching some of the exclusion borders (defined as three of the six faces of the parallelepiped defining the stack).

The object tagging algorithm is implemented in Java as an ImageJ plugin.^12^

### 2.4. Holes detection

**Figure 4:**
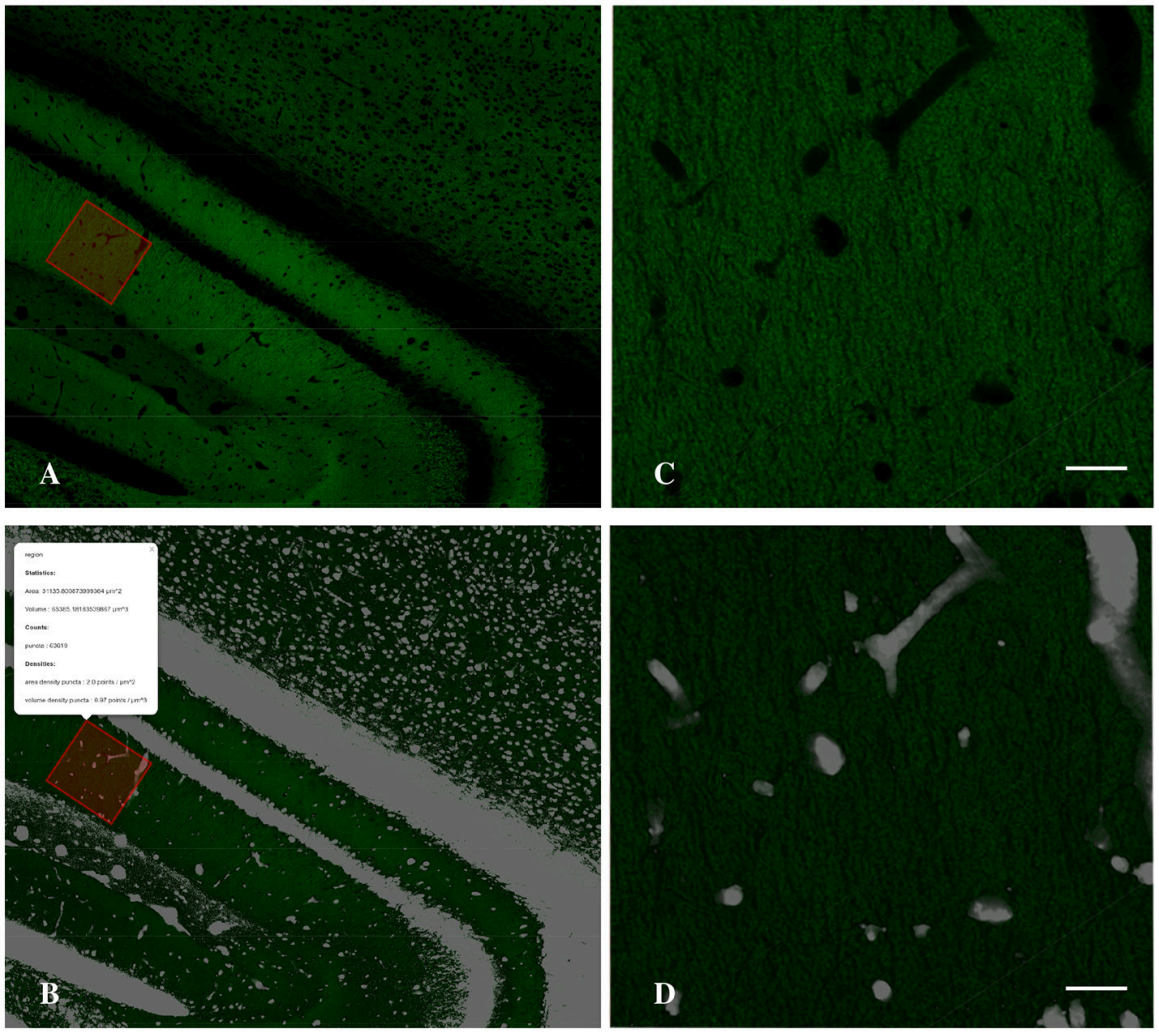
Detail of the Graphical Interface of MultiMap, showing CA1 hippocampal region **(A)** and the computed holes layer **(B)** and the result of the density estimation over a selected region (inset). **(C,D)** Higher magnification images of selected region shown in **A,B**, respectively. Scale bar is 20 μm.

To estimate the density of puncta in the neuropil it is essential to detect what we called the holes (cell bodies, the blood vessels and artifacts of the histological sections) in order to subtract their volume from the final density estimation. To detect the holes we invert the normalized gray-scale of the image, then we employ a median filter with a large radius to remove noise. Next, we set to zero every pixel with intensity less than a given threshold (*holesThr*) and to 1 the remaining pixels. The output is a binary (0–1) stack where pixels with values 0 belong to neuropil. To obtain a more compact representation (better suited both for visualization purposes and computational issues) we project the holes stack, summing along *z*-dimension, and we obtain a two dimensional holes mask where each pixel carries a non-negative integer that indicates how many of the slices are detected as not belonging to neuropil (See Figure 4).

### 2.5. Density estimation

Once the object detection and holes detection work-flows have been executed we obtain the centroids list and the holes mask stack. We are now able to obtain the density of puncta in whatever volume of the stack. Since in our case the depth of the stack is much smaller than the *x*-*y* dimensions, the volumes we are considering are defined by 2D regions in the *x*-*y* plane. The volume number density *d*(*R*) of puncta in a given volume define by a 2D region *R*, is estimated by following fraction:

$$d\left(R\right)=\frac{N_{s}\left(R\right)}{V\left(R\right)},$$

where *N*_*s*_(*R*) is the stereology unbiased estimation of the number of objects in *R*, obtained counting the centroids of the objects not touching some of the exclusion borders. *V*(*R*) is the volume of the solid with *x*-*y* section equal to *R* and depth equal to the stack's depth (δ), that is, *V*(*R*) = *A*(*R*) × δ where *A*(*R*) is the area of the polygon that defines the border of *R*.

The volume number density in the neuropil can similarly be estimated as

$$d_{n}\left(R\right)=\frac{N_{s}\left(R\right)}{V\left(R\right)-H\left(R\right)},$$

where *H*(*R*) is the volume of the holes computed by summing the values of the holes mask inside the region *R* and multiplying by the appropriate factor (the volume of a single voxel).

### 2.6. Validation

As pointed out in Carpenter et al. (2006) the direct comparison of image analysis software and procedures is difficult mainly because the results are influenced by how the softwares are tuned and, we suggest, also by the pre-processing steps. Usually the original images and the experts ground truth are not released making impossible to replicate the validation and to compare with new methods. In the bioimaging community it has become clear, as in other areas (see for example Lichman, 2013), the importance to benchmark and validate the image analysis methods over publicly available datasets with clear validation techniques and measures (Kozubek, 2016). Unfortunately to the present day there is no available benchmarking dataset for flourescent puncta segmentation. Thus we asked two experts to annotate a sample of the images as a ground truth. The data is publicly available in the Broad Bioimage Benchmark Collection^13^ (Ljosa et al., 2012) with instructions on how to perform the validation.

To prove the flexibility and performance of our method we also validated it using two image sets from the Broad Bioimage Benchmark Collection (Ljosa et al., 2012) (see Supplementary Material).

#### 2.6.1. vGlut-1 and vGat puncta detection

Each of the two experts was asked to detect the centroid of each puncta he/she sees in a bounded region and to mark it with a point in the original stack. We validated the procedure on subregions of 6 stacks for each marker for a total of 12 stacks analyzed, covering different distributions of puncta.

To match the results of the algorithm (obtained with parameters fixed as in Table 1) with the results each of the given experts we computed, apart from the raw number of objects detected, two scores: precision and recall. For that we consider an object as correctly detected if a point given by the expert lies inside one of the objects detected by the algorithm, obviously without associating in this way more than one point with the same object. Then, precision was computed as the fraction of correctly detected objects over the total number of objects detected by the algorithm. Recall is the fraction of correctly detected objects over the total number of objects identified by the expert. Both precision and recall measures ranged between 0 and 1 being 1 the best score value. The F1 score, that is, the harmonic mean of precision and recall, was used as a summarizing measure.

**Table 1:** Parameters used in the vGlut-1 and vGat experiments.

**parameter**	**value**
*r*_*min*_	4
*r*_*max*_	10
δ	1
*thrMethod*	Moments
ϕ	0.4
*minSize*	40
*toll*	0

**Figure 5:**
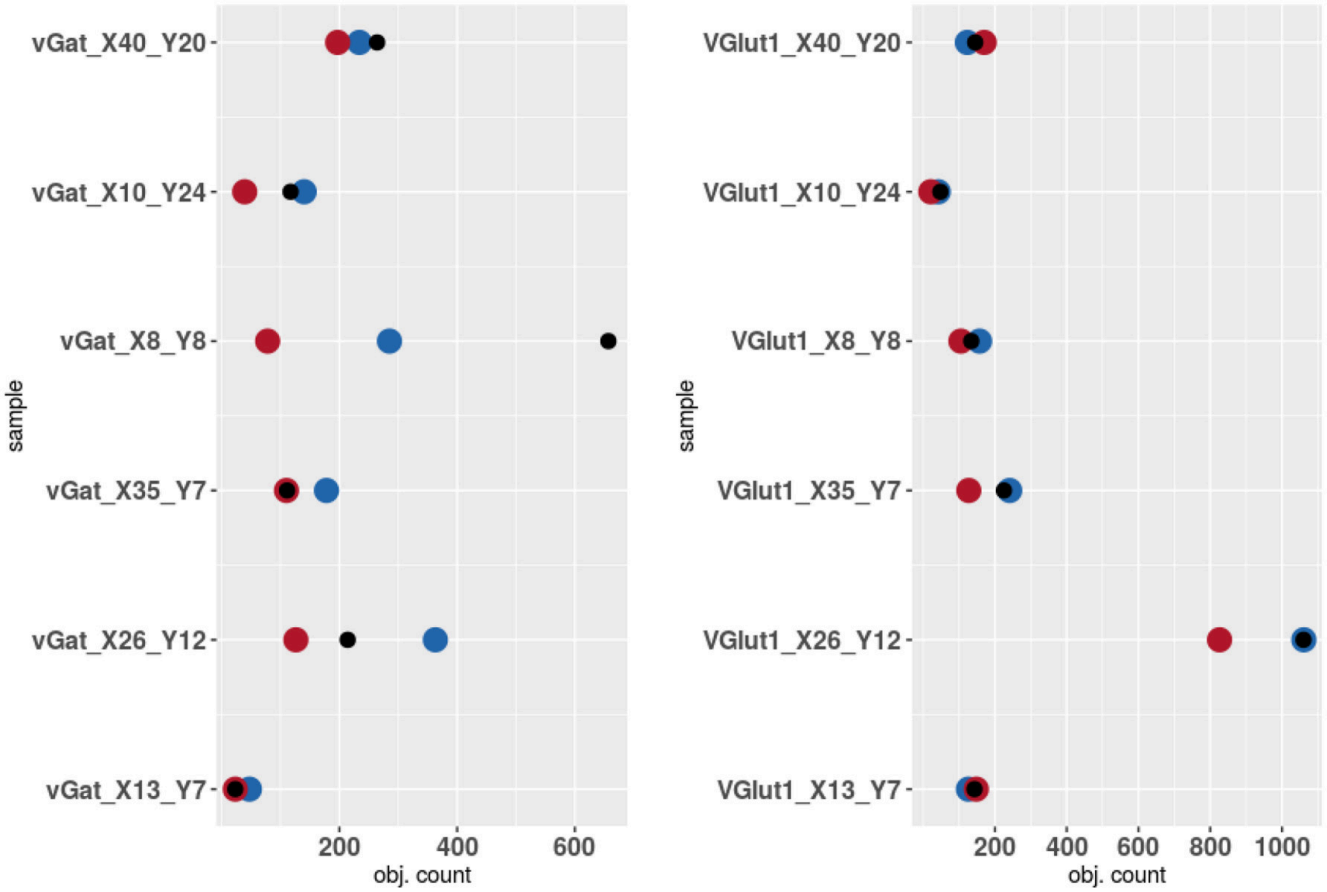
Results of the validation. Experts 1 and 2 are represented by red and blue points while algorithm counts are marked by black points.

The results of the validation are reported in Tables 3, 2 and presented graphically in Figures 5. As we can see the automatic method obtains a number of objects included between the two experts results in four of the six samples for the vGat marker and in five of the six samples for the vGlut-1 marker. Quantitatively we observe that for the vGat marker the average relative error between the experts and the mean count of the two experts is 0.38 while the average relative error between the automatic method and the mean count of the experts is 0.64, being this result due only to the error made by the algorithm in the sample vGAT_X8_Y8. The large error is possibly caused by the fact that the algorithm is able to see objects at a low intensity while the experts see just the brightest objects. The proposed algorithm incurs in similar errors in all stacks with very few or none puncta. The problem is due to the fact that in stacks with no puncta the algorithm detects noise (or other artifacts) as foreground since no real object is present to set a reference for the true foreground intensity. It is worth to observe that the relative error between the experts in the sample vGAT_X8_Y8 is among the highest showing that, without information on the stack position in the hippocampus, detections of puncta in regions with few or none objects is extremely difficult. This problem is absent in the neuropil. Thus, the holes detection procedure (section-2.4) could be used to filter-out areas where few or none puncta are expected. We preferred to report validation results for the segmentation and detection method alone to highlight such difficulties and limitations of the algorithm.

**Table 2:** Comparison with the two experts, vGlut-1 marker.

**Sample**	**Expert 1**	**Algorithm**	**Recall**	**Precision**	**F1 Score**
VGlut1_X13_Y7	148	143	0.59	0.62	0.61
VGlut1_X26_Y12	826	1060	0.60	0.49	0.53
VGlut1_X35_Y7	127	225	0.87	0.49	0.63
VGlut1_X8_Y8	105	134	0.71	0.55	0.62
VGlut1_X10_Y24	21	48	0.61	0.27	0.38
VGlut1_X40_Y20	170	145	0.54	0.63	0.58
**Sample**	**Expert 2**	**Algorithm**	**Recall**	**Precision**	**F1 Score**
VGlut1_X13_Y7	126	143	0.67	0.59	0.62
VGlut1_X26_Y12	1061	1060	0.60	0.60	0.60
VGlut1_X35_Y7	241	225	0.64	0.69	0.67
VGlut1_X8_Y8	157	134	0.58	0.68	0.63
VGlut1_X10_Y24	42	48	0.62	0.54	0.57
VGlut1_X40_Y20	123	145	0.67	0.57	0.62

**Table 3:** Comparison with the two experts, vGAT marker.

**Sample**	**Expert 1**	**Algorithm**	**Recall**	**Precision**	**F1 Score**
vGAT_X13_Y7	23	23	0.83	0.83	0.83
vGAT_X26_Y12	126	214	0.87	0.51	0.64
vGAT_X35_Y7	110	111	0.76	0.76	0.76
vGAT_X8_Y8	78	657	0.92	0.11	0.20
vGAT_X10_Y24	39	117	0.82	0.27	0.41
vGAT_X40_Y20	197	264	0.70	0.52	0.60
**Sample**	**Expert 2**	**Algorithm**	**Recall**	**Precision**	**F1 Score**
vGAT_X13_Y7	47	23	0.51	1	0.69
vGAT_X26_Y12	363	214	0.47	0.79	0.59
vGAT_X35_Y7	178	111	0.45	0.72	0.55
vGAT_X8_Y8	285	657	0.74	0.32	0.45
vGAT_X10_Y24	140	117	0.49	0.58	0.53
vGAT_X40_Y20	234	264	0.67	0.59	0.63

The automatic method obtains better results in the vGlut-1 marker. In this case the average relative error between experts and the mean count is 0.20 while the relative error between the algorithm and the mean count of the experts is 0.16.

**Figure 6:**
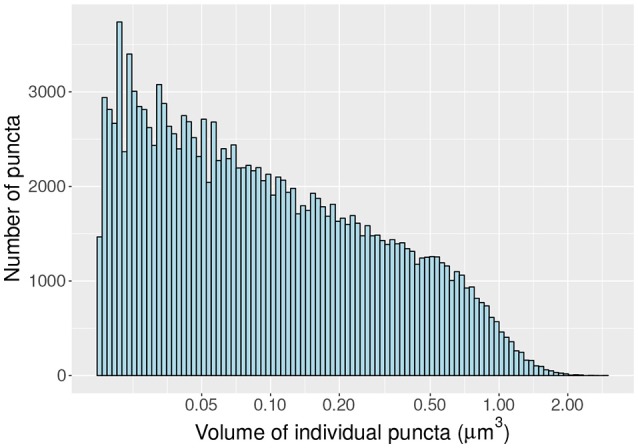
Histogram of volumes (log scale) of individal vGlut-1 puncta in the stratum radiatum of CA1.

As a proof of concept we compute the density and distribution of volumes (Figure 6) of individual vGlut-1 puncta in a region (surface 8.15 × 10^4^ μm^2; volume 1.71 × 10^5^ μm^3) of the stratum radiatum of CA1 extracted with the MultiMap interface. In this region the average density of vGlut-1 puncta was 0.92 puncta per μm^3^.

## 3. Discussion

The present paper presents a new approach to deal with large stacks of confocal microscopy images. It allows to easily explore, select and analyse structures (fluorescent puncta) present in the neuropil of large brain areas, that will be automatically 3D segmented and quantified. The possibility to create regions of interest, without any restrictions in its shape, within a large brain area is a great advantage of the tool compared to other available tools. Therefore, it will contribute to explore the large volume of data that is currently being generated to possibly develop large-scale computer simulation of the brain. Additionally, it permits a 3D segmentation and quantification (number and volumetry) of elements in a fully automated procedure. The segmentation procedure used in the present work (maximum of Laplacians of Gaussian filter and an automated object detection process, performed using a fast tag propagation algorithm) allowed for a fast and accurate 3D quantification of the numerous puncta present in the selected hippocampal region. The segmentation using maxLoGs has shown to perform well on images with low signal-to-noise ratio and very low resolution, in particular no previous deconvolution was applied to the stacks of images used in the validation. Since the segmentation is performed independently on every stack, some errors have to be expected when the algorithm is asked to detect objects where none are present. The segmentation and detection algorithm presented has been intentionally kept simple and with few tunable parameters. The aim was to implement a fast and easily usable method that could thus be embedded in the MultiMap interface.

The execution of the object detection algorithm in a single stack requires about 1.5 min of computing time^14^. Moreover, we observe that the time needed to process the stacks with the proposed algorithm is comparable to the time needed for the image acquisition. Ideally it would be possible to process very large regions of the brains, and even a whole brain; the algorithm could be applied to the obtained stacks of images while the confocal microscopy would extract the next set of stacks, thus not adding a significant amount of time to the whole process. Moreover, since the detections are performed independently on every stack the process can be easily parallelized.

Other available counting methods can also be easily implemented in MultiMap since it is linked to the widely used ImageJ software tool (Schneider et al., 2012). MultiMap has been developed as a basic GUI interface and all the capabilities are implemented as extension. We developed basic example extensions to compute and visualize region statistics^15^, to use GraphicsMagick^16^ image processing capabilities^17^ and to connect with R^18^ (R Core Team, 2017) through shiny (Chang et al., 2017). In the near future, we plan to implement spatial statistical procedure to test and fit spatial point process (Anton-Sanchez et al., 2014) and a three dimensional viewer. We built the MultiMap API^19^ in a way that permits the development and integration of future extensions. Moreover, we plan to include, through the ImageJ extension, various state of the art segmentation and image processing techniques that could be useful in different problems.

## Ethics statement

This study was carried out in accordance with recommendations for the proper care and use of laboratory animals and under the authorization of the regulations and policies governing the care and use of laboratory animals (EU directive no. 86/609 and Council of Europe Convention ETS1 23, EU decree 2001-486, Spanish Royal Decree 53/2013 and Law 6/2013 and Statement of Compliance with Standards for Use of Laboratory Animals by Foreign Institutions no A5388-01, National Institutes of Health, USA). In addition, the protocol was approved by the Ethical Animal Commission for San Pablo CEU University's Animal Facility: CEBA USP-CEU (Madrid, Spain).

## Author contributions

GV: developed the object detection procedure and implemented it in Java as an ImageJ extension, he also designed and implemented the MultiMap application and the related libraries, moreover he carried out the computational experiments and the validations. RB-P: contributes to the design of the object detection and validation procedure. AM: acquired the data and validation of the objects detection. AK: participates in the validation of the objects detection. The manuscript was jointly written by all the authors.

### Conflict of interest statement

The authors declare that the research was conducted in the absence of any commercial or financial relationships that could be construed as a potential conflict of interest.

## Footnotes

^1^https://computationalintelligencegroup.github.io/MultiMap-documentation/

^2^Download the configuration.json file from http://cajalbbp.cesvima.upm.es/maps/hippocampus_mouse_HBP21_id4_40_vglut1/ and load it into MultiMap

^3^http://leafletjs.com/

^4^https://github.com/gherardovarando/leaflet-map-builder

^5^http://json.org/

^6^For an extensive list see https://github.com/gherardovarando/leaflet-map-builder

^7^http://wiki.openstreetmap.org/wiki/Tiles

^8^https://www.openmicroscopy.org/bio-formats/

^9^Using Map Tools ImageJ plugin https://github.com/ComputationalIntelligenceGroup/Map_tools

^10^https://github.com/ComputationalIntelligenceGroup/obj_detection_toolset

^11^https://github.com/ComputationalIntelligenceGroup/MaxLoGs

^12^https://github.com/ComputationalIntelligenceGroup/ObjCounter

^13^https://data.broadinstitute.org/bbbc/ dataset to appear soon.

^14^Experiments executed on a machine equipped with Intel Core i7-5820k, 3.30 GHz × 12 and 64 GB of RAM, the algorithm uses just one core and approximately 1 GB of memory.

^15^https://github.com/gherardovarando/regionstatsextensions

^16^www.graphicsmagick.com

^17^https://github.com/gherardovarando/GraphicsMagickExtension

^18^https://github.com/gherardovarando/RShinyExtension

^19^see MultiMap user guide https://computationalintelligencegroup.github.io/MultiMap-documentation/
