# Supplementary material for: MultiMap: A Tool to Automatically Extract and Analyse Spatial Microscopic Data From Large Stacks of Confocal Microscopy Images
Source: Front Neuroanat. 2018 May 23;12:37. doi: 10.3389/fnana.2018.00037 (PMC5974206; doi:10.3389/fnana.2018.00037)
Supplement: Supplementary file 1 [file Data_Sheet_1.PDF]

Supplementary Material: MultiMap: A tool to automatically extract and analyze spatial microscopic data from large stacks of confocal microscopy images

1 SUPPLEMENTARY VALIDATIONS

We report here the additional validations performed over two publicly available image sets. Both image sets belong to the Broad Bioimage Benchmark Collection.[1] The validation measures and methods are fully described in the image sets' description page, giving an unique way to benchmark every algorithm.

1.1 Human HT29 colon-cancer cells

The image set selected is BBBC001v01[2] (Carpenter et al., 2006). It consists of 6 fields of view and the cells have been treated with an RNA interference reagent (Rock1 1885 k27). The samples were stained with Hoechst 33342, pH3, and phalloidin. Hoechst 33342 is a DNA stain that labels the nucleus. Only the images of the Hoechst 33342 channel are included, as the other channels are not useful for counting cells. The images were acquired at the Whitehead-MIT Bioimaging Center on a Cellomics ArrayScan. The image size is 512×512 pixels. The ground truth is given as the cell count in each of the images by two human counters.

We apply our method with the parameters fixed as in Table S1, found by a non expert in biology with trials and errors over the first image of the dataset (parameter ϕ is irrelevant given that the images are in 2D).

The comparison was carried out as explained in the image set description: we computed for each image the absolute difference between the algorithm's count and the average of the humans' counts, then we divided by the latter to obtain the deviation from ground truth. The two human observers vary by 11% for the image set, while Carpenter et al. (2006) obtained a deviation of 6.2% and our method achieved 1.3% of deviation from the ground truth.

[1] https://data.broadinstitute.org/bbbc/

[2] https://data.broadinstitute.org/bbbc/BBBC001/

Table S1. Parameters used in Human HT29 colon cancer cells.

parameter	value
r_{min}	2
r_{max}	10
δ	1
thrMethod	Triangle
ϕ	$--$
minSize	60
toll	0

Table S2. Parameters used in Drosophila Kc167 cells, the sample of application is shown in parenthesis.

	value		
parameter	{*48* , *mad2, nodsRNA*}	*340*	*Anillin*
r_{min}	3	10	8
r_{max}	9	25	20
δ	2	3	2
thrMethod	IsoData		
ϕ	$--$		
minSize	20	20	20
toll	1	2	4

1.2 Drosophila Kc167 cells

We used the image set BBBC002v1[3] (Carpenter et al., 2006). The data comprises five different samples of Drosophila melanogaster Kc167 cells stained with Hoechst 33342. The last sample (labeled *nodsRNA*) is of wild-type cells. Each of the other four samples (labeled *48*, *340*, *Anillin*, and *mad2*) had a different gene knocked down by RNAi. The sample preparation is described in more detail by Carpenter et al. (2006). The ground truth is provided as the number of cells in each image counted by two different experts.

The object detection algorithm were applied with the parameters set as described in Table S2. We had to vary the parameters in two of the samples given the large differences in cells' shapes and sizes.

To compare the algorithm we followed the instructions given in the image set. We computed for each sample the algorithm's mean cell count over the 10 images of the sample. Next, we calculated the absolute difference between this mean and the average of the humans' counts for the sample and we divided by the latter to obtain the deviation from ground truth. We reported then the mean value over the five samples. The two experts vary by 16%, Carpenter et al. (2006) reported a 17% deviation using their method, while our algorithm obtained a 4.6% deviation.

REFERENCES

Carpenter, A. E., Jones, T. R., Lamprecht, M. R., Clarke, C., Kang, I. H., Friman, O., et al. (2006). CellProfiler: Image analysis software for identifying and quantifying cell phenotypes. *Genome Biology* 7, R100

[3] `https://data.broadinstitute.org/bbbc/BBBC002/`
